# Supplementary material for: Complexome profiling of the Chlamydomonas psb28 mutant reveals TEF5 as an early PSII assembly factor
Source: Plant Cell. 2025 Mar 17;37(6):koaf055. doi: 10.1093/plcell/koaf055 (PMC12164590; doi:10.1093/plcell/koaf055)
Supplement: koaf055_Supplementary_Data [file koaf055_supplementary_data.zip › psb28_interactive.zip!/plots/intensity/qualityPlots/peptides/PHB2_Cre12.g519350.t1.2.html]

### Cre12.g519350.t1.2

Trivial name: PHB2  
SeqCoverage [median]: 66.30  
Mass: 31.25 kDa  
  
 

| Sequence | MissedCleavage | Mass [Da] | wtIntensity | muIntensity | Score |
| --- | --- | --- | --- | --- | --- |
| AIIFDR | 0 | 733.41 | 0.00 | 3290.61 | 77.75 |
| ANPETAAVAINVLNK | 0 | 1523.83 | 5310.11 | 0.00 | 182.71 |
| AVVAQYNAEQLITQR | 0 | 1702.90 | 5226.05 | 15657.90 | 191.79 |
| DLQMVNMSLR | 0 | 1205.59 | 878.65 | 4312.33 | 105.98 |
| FVVMKAEQER | 1 | 1235.63 | 3155.37 | 4777.32 | 86.62 |
| GVLDEPVGEGTHFR | 0 | 1511.74 | 3929.63 | 7167.22 | 172.09 |
| ILSKPDEPR | 1 | 1053.58 | 1944.27 | 3856.85 | 64.04 |
| KIEAAKDVAETMSK | 2 | 1519.79 | 8843.84 | 3174.26 | 80.51 |
| LISDATK | 0 | 746.42 | 540.03 | 0.00 | 79.15 |
| NAAIIKAEGESEAAK | 1 | 1500.78 | 5579.84 | 8703.82 | 99.57 |
| NVVYLPNTGNMLMQVNPNQ | 0 | 2145.03 | 0.00 | 1844.45 | 85.29 |
| QVAEQDAER | 0 | 1044.48 | 2691.45 | 0.00 | 98.03 |
| QVAEQDAERAK | 1 | 1243.62 | 1155.45 | 375.97 | 102.53 |
| SISSVTGTK | 0 | 878.47 | 0.00 | 8661.02 | 69.83 |
| SVRESLMAR | 1 | 1047.55 | 528.90 | 0.00 | 101.97 |
| VLPSIGNEVVK | 0 | 1153.67 | 1148.50 | 10271.41 | 80.69 |
|  |  |  | 40932.1 | 72093.1 |  |
